# Accounting for horizontal gene transfers explains conflicting hypotheses regarding the position of aquificales in the phylogeny of Bacteria

**DOI:** 10.1186/1471-2148-8-272

**Published:** 2008-10-03

**Authors:** Bastien Boussau, Laurent Guéguen, Manolo Gouy

**Affiliations:** 1 Université de Lyon; Université Lyon 1; CNRS; INRIA; Laboratoire de Biométrie et Biologie Evolutive, 43 boulevard du 11 novembre 1918, Villeurbanne F-69622, France

## Abstract

**Background:**

Despite a large agreement between ribosomal RNA and concatenated protein phylogenies, the phylogenetic tree of the bacterial domain remains uncertain in its deepest nodes. For instance, the position of the hyperthermophilic Aquificales is debated, as their commonly observed position close to Thermotogales may proceed from horizontal gene transfers, long branch attraction or compositional biases, and may not represent vertical descent. Indeed, another view, based on the analysis of rare genomic changes, places Aquificales close to epsilon-Proteobacteria.

**Results:**

To get a whole genome view of *Aquifex *relationships, all trees containing sequences from *Aquifex *in the HOGENOM database were surveyed. This study revealed that *Aquifex *is most often found as a neighbour to Thermotogales. Moreover, informational genes, which appeared to be less often transferred to the *Aquifex *lineage than non-informational genes, most often placed Aquificales close to Thermotogales. To ensure these results did not come from long branch attraction or compositional artefacts, a subset of carefully chosen proteins from a wide range of bacterial species was selected for further scrutiny. Among these genes, two phylogenetic hypotheses were found to be significantly more likely than the others: the most likely hypothesis placed Aquificales as a neighbour to Thermotogales, and the second one with epsilon-Proteobacteria. We characterized the genes that supported each of these two hypotheses, and found that differences in rates of evolution or in amino-acid compositions could not explain the presence of two incongruent phylogenetic signals in the alignment. Instead, evidence for a large Horizontal Gene Transfer between Aquificales and epsilon-Proteobacteria was found.

**Conclusion:**

Methods based on concatenated informational proteins and methods based on character cladistics led to different conclusions regarding the position of Aquificales because this lineage has undergone many horizontal gene transfers. However, if a tree of vertical descent can be reconstructed for Bacteria, our results suggest Aquificales should be placed close to Thermotogales.

## Background

In the study of evolution, as in any scientific endeavour, progress relies on the comparison of hypotheses with respect to how well these succeed in accounting for a range of observed data. In phylogenetics, a given tree, a hypothesis, is confronted with trees inferred using other data; resulting incongruences are then explained by a methodological artefact, or the inability of a single tree to properly depict the evolution of the biological entities under consideration. The large agreement between the ribosomal RNA (rRNA) bacterial phylogeny and phylogenies built from a concatenated set of protein sequences was therefore a strong piece of evidence that the tree of life could be solved [1]. For instance, protein phylogenies confirmed the monophyly of most rRNA-defined bacterial phyla. Similarly, Aquificales are found close to Thermotogales both in trees built from rRNA and from concatenated proteins. However, the position of the Aquificales clade within the phylogeny of Bacteria has often been questioned on the ground of single gene phylogenies, phylogenies built from gene or domain content [2], and supposedly rare genomic changes such as insertions-deletions [3-8]. Strikingly, many of these analyses are congruent with each other and suggest that Aquificales might be more closely related to Proteobacteria than to Thermotogales. This new view has been adopted in recent scenarios that explain the whole evolution of life on earth [9], so it is important to our understanding of bacterial evolution that the puzzling phylogenetic problem of the position of Aquificales within the bacterial phylogeny gets solved.

Species phylogenies built from the comparison of gene sequences suffer from two major limitations: on one side the true gene trees may differ from the species trees, and on the other side, the signal contained in the gene sequences might be too weak or too complex to be correctly interpreted by bioinformatics methods. Gene trees will differ from species trees in cases of hidden paralogy, closely spaced cladogenesis events or horizontal gene transfers (HGT). This last phenomenon is particularly relevant to the present study, as gene transfers are frequent among prokaryotes. Phylogeneticists therefore often only consider informational genes, involved in the processes of transcription, translation and replication, which appear to be less prone to HGTs over broad distances than other genes, named operational [10]. The second limitation, that of a phylogenetic signal so blurred or buried that tree reconstruction methods fail to recover the true tree, may come from a saturated history of mutations (long branch attraction, [11,12]) or compositional biases [13,14]. Both pitfalls are likely to affect genes used to reconstruct the bacterial phylogeny, because Bacteria possibly date as far back as 3.5 billion years ago [15], and because they display a great diversity in their genomic characteristics as well as in their ecological niches. More specifically, Aquificales may be placed close to Thermotogales not because they last diverged from them, but because they share a common ecological niche, *i.e*. they are both hyperthermophilic, which led both their rRNA [16] and their protein sequences [17] to adapt to high temperatures. Sequence similarities between these two clades would therefore be the result of convergences due to identical selective pressures, not the result of common descent. Consequently, recovering the bacterial species tree and clarifying the relations between hyperthermophilic organisms from comparison of gene sequences is a difficult task, and has led several authors to search for more reliable informative characters.

Such characters are cell-structural features, or of a genomic nature: "rare genomic changes" [18], such as gene fusion/fission or insertion-deletions (indels), and gene or domain presence/absence. The main assumption concerning all these characters is that they are nearly immune to convergence: to be informative, a given character, morphological or genetic, should only arise once. To our knowledge, this assumption has never been thoroughly tested. The genomic characters further depend on the identification of orthologous genes in different genomes, and consequently are subject to the pitfall of horizontal gene transfers. Here again, this weakness is of particular interest to our study, since both Aquificales and Thermotogales seem to be particularly prone to exchanging genes with other bacterial species [19,20].

Therefore it appears that both approaches – sequence phylogenies and character cladistics – are potentially hindered by defaults whose magnitude is sufficient to question their conclusions. As in the case of the phylogenetic position of Aquificales their conclusions diverge, a detailed study might clarify which approach has suffered most from its drawbacks.

In this report, we used the HOGENOM [21] database to survey the phylogenetic neighbourhood of *Aquifex*. This database contains families of homologous genes from complete genome sequences with associated sequence alignments and maximum likelihood phylogenetic trees. The automatic survey of all trees containing sequences from *Aquifex *in the HOGENOM database reveals that *Aquifex *is most often found as a neighbour to Thermotogales. When genes are separated into informational and non-informational genes we find that genes from the former category seem to be less transferred than non-informational ones. To this end, neighbour clades for each gene from *Aquifex *were counted, separately for informational genes and for operational genes, yielding two distributions. Then for each of the two distributions, Shannon's index of diversity was computed [22]. This index measures whether the genes are evenly distributed among all possible neighbourhoods or whether a specific vicinity dominates. We find that the index value is significantly different between the two distributions: among informational genes, one neighbourhood, between Aquificales and Thermotogales, tends to dominate the distribution much more than in operational genes. This shows that there is one dominating phylogenetic signal among informational genes, and much less among operational genes, which is consistent with the idea that operational genes experience more frequent HGT events than informational genes.

To study the impact of saturation and compositional heterogeneity on the position of Aquificales, we concatenated a large dataset of putatively orthologous proteins from a wide range of bacterial species (Additional file 1). A phylogenetic tree was built, and then taken as a reference to test for the position of Aquificales: Aquificales were first removed from the tree, and then re-introduced in the topology in all possible positions. Site likelihoods were computed for all these positions, which allowed for the identification of sites favouring a given topology. Two phylogenetic hypotheses were found to be significantly more likely than the others: the most likely hypothesis placed Aquificales as a neighbour to Thermotogales, and the second one placed Aquificales with epsilon-Proteobacteria. We characterized the genes that supported each hypothesis, and found that differences in rates of evolution or in amino-acid compositions could not explain the presence of two dominating phylogenetic signals in the alignment. However, evidence for a large Horizontal Gene Transfer between Aquificales and epsilon-Proteobacteria was found. These findings suffice to explain why methods based on concatenated informational proteins and methods based on character cladistics led to different conclusions, and suggest that the vertical signal in the genomes of Aquificales, *i.e*. the portion of the genome most likely to have been inherited through descent and not through HGT, relates them to Thermotogales.

## Results and discussion

### A whole genome view of *Aquifex *relationships

**Figure 1:**
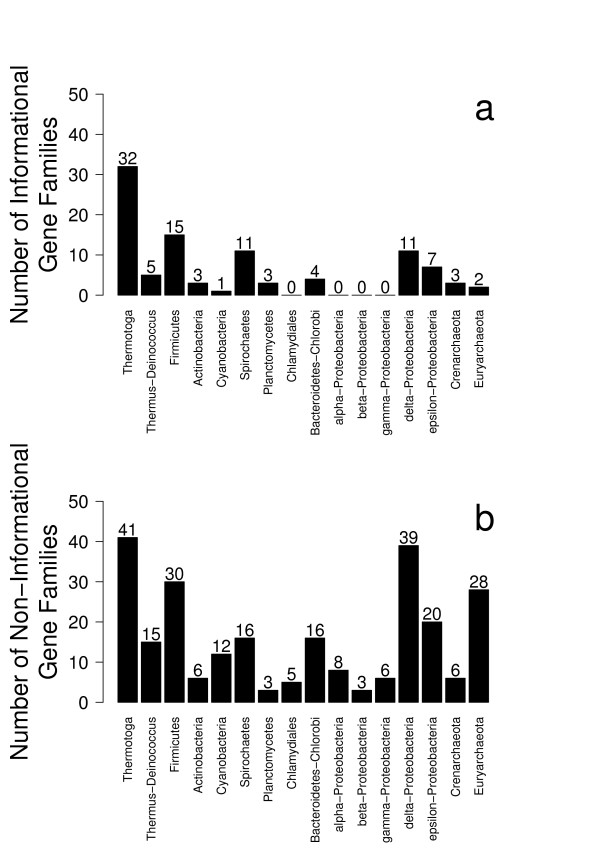
**Phylogenetic relationships of *Aquifex *genes according to the HOGENOM database.** a: Informational genes. b: Non-informational genes.

For each gene tree containing sequences from *Aquifex aeolicus *in the HOGENOM database, the identity of the group of sequences neighbouring *Aquifex *was recorded. This gave counts of *Aquifex *genes found close to Thermotogales, Firmicutes, epsilon-Proteobacteria, among others. Cases where *Aquifex *genes were found close to a non-monophyletic group of species were discarded, which left 578 gene trees. Among these, *Thermotoga *is found as *Aquifex's *closest neighbour 98 times, epsilon-Proteobacteria are found 44 times, delta-Proteobacteria 84 times, Firmicutes 71, Thermus-Deinococcus 39, Euryarchaeota 74 (see Fig. 1). In view of such a distribution, it is difficult to argue in favour of any particular relationship: Horizontal Gene Transfers appear so pervasive that no signal emerges as clearly dominant. However, HGTs may not affect all types of genes with similar frequencies. It has been proposed that genes that are related to the universal processes of transcription, translation and replication and known as "informational genes" may be less transferred than "operational genes", involved in metabolism for instance [10].

We therefore separated HOGENOM protein families into informational and non-informational gene families. Fig. 1a shows that among informational genes, the genes placing *Aquifex *close to *Thermotoga *(32 genes) are twice more numerous than the genes favouring the second best alternative hypothesis, *i.e*. the vicinity of Firmicutes (15 genes). On the contrary, among operational genes (Fig. 1b), differences between various hypotheses are much narrower: *Thermotoga *is *Aquifex's *neighbour in only two more cases than delta-Proteobacteria, 11 more cases than Firmicutes, and 13 more cases than Euryarchaeota. To quantify this comparison, Shannon's index of diversity was measured for both sets of genes. This index measures how evenly distributed observations are among categories [22]: the higher the index, the more even the distribution; conversely, the lower the index, the more a few categories dominate. Shannon index values were 2.07 for informational genes, and 2.49 for operational genes (significantly different according to a t-test, p-value < 0.001; a Pearson χ^2 ^test between the two distributions is also significant, p-value < 10^-20^), which means that operational genes are significantly more evenly distributed among the various neighbour groups than informational genes. The distributions depicted in Figs 1a and 1b result from a mixture of lack of phylogenetic resolution at the single-gene level and of HGT events. But the difference between them strongly suggests that operational genes have been horizontally transferred more often than informational genes, which is consistent with the fact that Euryarchaeota are almost never found as neighbour to *Aquifex *in informational genes (2%), but often found in operational genes (11%). Interestingly, for both sets of genes, epsilon-Proteobacteria are not one of the most frequent *Aquifex *neighbours, as they are less frequent than *Thermotoga*, Firmicutes, and delta-Proteobacteria. For operational genes, they are even less frequent than Euryarchaeota. These results thus do not support the hypothesis that Aquificales are epsilon-Proteobacteria [4]. However, if all Proteobacteria are to be counted as a single clade, the vicinity of *Aquifex *with Proteobacteria becomes a high-scoring hypothesis: *Aquifex *is most closely related to a Proteobacterium with 18 informational genes and 76 non-informational genes. According to operational genes, if anything, *Aquifex *would be a Proteobacterium, as almost twice more genes place it with Proteobacteria than with *Thermotoga *(76 for Proteobacteria against 41 for *Thermotoga*); according to informational genes, *Aquifex *is close to *Thermotoga*, as almost twice more genes place it with *Thermotoga *than with Proteobacteria (18 for Proteobacteria against 32 for *Thermotoga*). However, considering all Proteobacteria as a single clade artificially groups a variety of different histories under the same hypothesis. It is thus more likely that the high frequency of close relationships between *Aquifex *and *Thermotoga *among informational genes reflects vertical descent, and that the scattered distribution of *Aquifex *closest homologs among operational genes results from frequent horizontal transfers to or from the *Aquifex *lineage.

Furthermore, this whole genome analysis may suffer from compositional biases or long branch attraction. Consequently, a subset of carefully chosen genes was concatenated and used to assess the importance of potential artefacts: first a tree of the Bacteria was built, and then, using this tree as a scaffold, the influence of saturation and compositional biases on the position of Aquificales was estimated.

### Bacterial phylogeny obtained from a concatenated set of putatively orthologous genes

**Figure 2:**
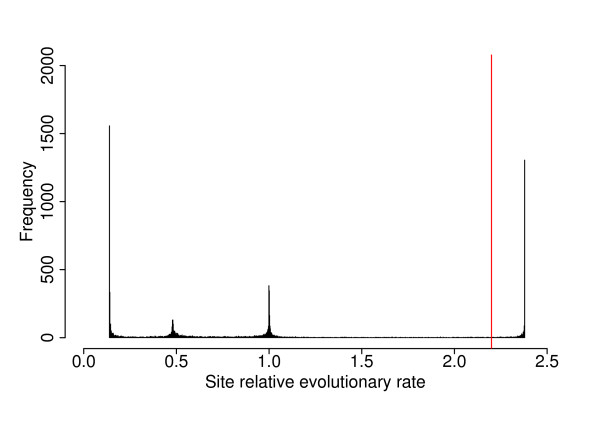
**Distribution of the site relative evolutionary rates.** Rates were estimated using a 4 class discretized gamma distribution. The 4 peaks correspond to the rates associated to each class. The vertical red line corresponds to the threshold above which sites have been discarded due to their high evolutionary rate.

Fifty-six genes that were nearly universal in Bacteria and present as single copy in most genomes were concatenated (see Methods). Genes that showed a transfer between Bacteria and Archaea had previously been discarded because a gene showing evidence of a transfer between very distantly related organisms might be especially prone to be transferred among species of the same domain. Some of the 56 remaining genes may still have undergone a transfer, and concatenating them may lead to spurious results. Usually, transferred genes are discarded before gene concatenation [23,24]. Here, we first checked for possible tree building biases resulting from composition or evolutionary rate effects before proceeding to an analysis designed to specifically identify genes that may have been transferred between *Aquificales *and other species. PhyML was used to build a starting phylogeny based on the concatenated protein alignments, using the JTT model and a gamma law discretized in four classes to account for variation in the evolutionary rates. The discretized gamma law [25] is widely used because of its mathematical convenience, not as a precise model of the evolutionary rates of protein sequences. Therefore it is expected that some sites are not properly modelled when this approximation is made. To estimate how sites were modelled by the discretized gamma law, we plotted the distribution of expected relative evolutionary rates across sites (Fig. 2) as found by BppML. This distribution shows four peaks, each corresponding to the rate of a particular class. The two largest peaks are at the limits of the distribution: they comprise both sites whose rate is properly approximated by one of the two extreme evolutionary rates, but also sites whose rate would be smaller or larger, if the discretized gamma law was able to provide a convenient rate. For instance, the leftmost peak contains sites properly modelled by a relative rate of ~0.2, but also sites evolving more slowly, such as constant sites. *Per se*, improperly modelling constant sites probably does not lead to biased phylogenetic estimations; however underestimating the evolutionary rate of some fast-evolving sites (and this may be a by-product of improper modelling of constant sites) will lead to an underestimation of the convergence probability. Such misspecified modelling is therefore a potential cause for long branch attraction, as underlined in another context [26]. We consequently decided to conservatively discard sites whose evolutionary rate was above the arbitrary threshold of 2.2 (red line, Fig.2), in the hope of reducing risks of reconstruction artefacts. The resulting alignment contains 10,000 sites, and has been submitted to an additional reconstruction through PhyML, with a bootstrap analysis based upon 200 replicates.

**Figure 3:**
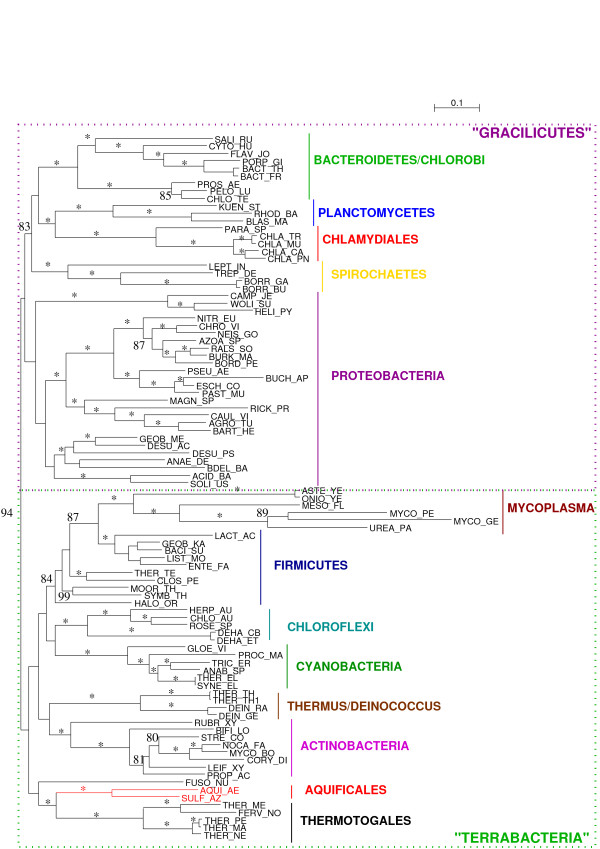
**Unrooted phylogenetic tree of Bacteria.** This tree was obtained after discarding all sites with evolutionary rate predicted to be above 2.2. Stars indicate branches with 100% bootstrap support (200 replicates). Bootstrap supports between 80% and 100% are shown, bootstraps below 80% have been removed for clarity. Aquificales are represented in bright red. Names of major groups are according to the NCBI taxonomy. Gracilicutes and Terrabacteria, two recently proposed superclades, are shown as dashed frames, and their names are between quotation marks to mark their unconsensual status.

Our tree comprises 94 bacterial species, spanning as exhaustively as currently possible the diversity of Bacteria (Fig. 3). The resulting topology is in good agreement with rRNA trees [27], recently published concatenated-protein phylogenies [28,29], as well as supertree phylogenies [30]. In particular, we do recover the clade named "Terrabacteria" by Battistuzzi and co-workers, as well as the clade named Gracilicutes by Cavalier-Smith [7], separated with a high bootstrap support (BS 94%). It is interesting to note that these three recent bacterial phylogenies all recover these two clades, which suggests that the global picture of bacterial evolution might be slowly unveiling. The "PVC supergroup" (Planctomyces-Verrucomicrobia-Chlamydiales, [31]) seems to find a confirmation in our phylogeny where Planctomycetes and Chlamydiales are grouped with 100% BS. Many similarities are also found with the phylogeny proposed by Ciccarelli and co-workers [32], or the supertree obtained by Beiko, Harlow and Ragan [33], such as the monophyly of Proteobacteria, and the grouping of Aquificales with Thermotogales.

However, many deep nodes do not obtain high bootstrap supports. Two avenues might help fully resolve the bacterial phylogeny: further increase the number of phylogenetic markers, and improve the interpretation of the phylogenetic signal through the development of new models of evolution. Such models would ideally be able to deal with compositional heterogeneity, and would safely handle saturation. As there is no efficient program with these properties, we have chosen to filter out saturated sites to try and diminish compositional heterogeneity.

We have already attempted to remove the most saturated sites. To assess the impact of compositional heterogeneity, we performed Bowker's tests for symmetry in the evolutionary process on the whole alignment [34,35]. Bowker's test relies on the comparison of two sequences against each other, therefore 94*93/2 = 4371 tests can be done on our alignment. Among these 4371 tests, 3826 reject symmetry at the 5% level: though we have made no effort to alleviate the multiple tests problem, compositional heterogeneity might be an important issue for the reconstruction of bacterial phylogeny. Species that show the most biased amino-acid usage, *i.e*. that fail the highest numbers of Bowker's tests, include first AT-rich species (*Buchnera aphidicola*, *Borrelia burgdorferi*), then GC-rich species (*Thermus Thermophilus*) and finally hyperthermophilic species (data not shown). This is in agreement with results based on a multivariate analysis of proteome composition [36], where the GC content of the genome was found to be the major factor influencing amino-acid composition, before thermophily.

**Figure 4:**
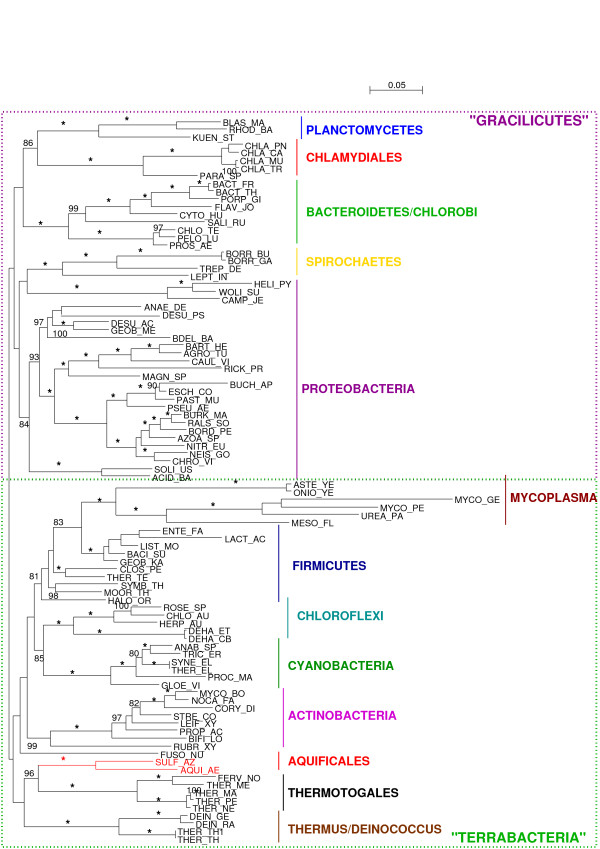
**Unrooted phylogenetic tree obtained from 56 genes of Bacteria based on the recoded alignment.** Labels as in Fig. 3.

To try and limit the influence of compositional bias, we recoded the concatenated protein alignment in 4 states based on the physico-chemical properties of the amino-acids [37]. Such a recoding is expected to reduce the risk of long branch attraction artefact as well as compositional bias by decreasing the number of homoplasies. Accordingly, after the recoding, 2818 tests reject symmetry: the recoding seems to have diminished compositional bias at least in 1008 cases, but clearly has not permitted to fully erase heterogeneity. The tree we obtain on the recoded alignment (Fig. 4) is very similar to the previous tree (Fig. 3), with Gracilicutes separated from Terrabacteria (BS 76%). Interestingly, Aquificales are still found as a sister group of Thermotogales with a high bootstrap support (96%), and Thermus-Deinococcus also clusters with these hyperthermophilic organisms, although the bootstrap support is negligible (36%). The grouping of the photosynthetic lineages Chloroflexi and Cyanobacteria gains support through the recoding, with a BS of 85% on the recoded alignment against 77% on the original alignment. So does the clustering of these two photosynthetic lineages with another lineage that contains photosynthetic organisms, the Firmicutes: from 63% on the original alignment, the BS increases to 73% with the recoded alignment. The grouping of these three photosynthetic lineages appears as an appealing hypothesis, but certainly requires further inquiry, especially since horizontal gene transfers are thought to have been part of the evolution of photosynthesis [38]. Strikingly, Spirochaetes were found to group with Chlamydiales, Planctomycetes and Bacteroidetes/Chlorobi with a high bootstrap support (83%) on the original alignment, but grouped with epsilon-Proteobacteria on the recoded alignment (bootstrap support: 18%), which shows that recoding can impact tree reconstruction. Overall, the average bootstrap support is 87.1%, not significantly lower than the average support for the original alignment (90.3%, p-value = 0.065 with a Student paired t-test, p-value = 0.154 with a Wilcoxon signed rank test). This supports the conclusion of Susko and Roger [39] that recoding does not lead to a substantial loss of information.

As the trees obtained on the recoded and original alignments are in strong agreement, we conclude that we obtain a fairly robust Bacterial tree, and that the clustering of Aquificales and Thermotogales does not seem due to saturation or compositional artefacts. However, since more than 50% of Bowker's tests reject symmetry on the recoded alignment, considerable compositional heterogeneity has escaped the 4-state recoding, and this analysis cannot entirely rule out the hypothesis that Aquificales and Thermotogales are attracted by compositional biases. Nonetheless, the addition to the concatenated alignment of sequences from two free-living epsilon-Proteobacteria, *Sulfurovum *NBC37-1 and thermophilic *Nitratiruptor *SB155-2 [40], does not affect this grouping either (see additional file 2). Thus the Aquificales-Thermotogales grouping does not seem to result from compositional biases.

### Does the Thermotogales-Aquificales cluster come from a reconstruction artefact?

The topology that is found without Aquificales using PhyML with the same parameters is perfectly congruent with the tree obtained with Aquificales. Taking therefore as reference the tree without Aquificales, we tested all possible positions for this group in the bacterial tree. The most likely position was as found by the tree search heuristics, with Thermotogales. The second most likely position was very close, at the base of a clade comprising both Thermotogales and Fusobacterium, and the third most likely position was with epsilon-Proteobacteria, the only placement not rejected at the 5% level according to an AU test [41] as implemented in Consel [42] (p-value = 0.062). Because the AU test is based on a multiscale RELL bootstrap procedure, the fact that the second most likely hypothesis is rejected by the AU test at 5% while the third is not suggests that sites of high likelihood scores are the same in the two first hypotheses, but are different from the sites of high likelihood scores in the third hypothesis. Consequently two contrasting signals can be found in the data, coming from different sites in the alignment, that support the two currently prevailing phylogenetic hypotheses for Aquificales, one based on rRNA trees, and the other heralded by Cavalier-Smith [4]. We decided to further analyse the nature of the signal that favoured each of these two placements, through a gene-wise analysis.

We built phylogenetic trees for each of our 56 genes with PhyML. Among these 56 trees, 11 place Aquificales close to Thermotogales (T genes), and only two place Aquificales close to epsilon-Proteobacteria (E genes). We compared these two sets of genes, with respect to rates of evolution and amino-acid composition, to see whether one signal is the result of a long branch attraction or of a compositional bias.

First, we computed the sum of the branch lengths for each tree in our two datasets, and computed an average branch length for each dataset. The average branch length was 0.163 for T genes, and 0.131 for E genes, which is not significantly different according to an unpaired t-test (p-value: 0.145). The discrepancy between the two datasets does not seem to be explainable by a long branch attraction artefact.

Second, the position close to Thermotogales might be favoured because of convergences instead of common descent: as written above, both Thermotogales and Aquificales are hyperthermophilic organisms, so their sequences are subject to partly similar selective pressures. Through the analysis of many completely sequenced genomes, Zeldovich and co-workers [17] have found a positive correlation between the proteome content in amino acids IVYWREL and the organism optimal growth temperature. As hyperthermophilic bacteria and archaea are not monophyletic, this suggests that there exists a selective pressure to increase the IVYWREL content in organisms that thrive best at high temperatures. If we find a higher proportion of the amino-acids IVYWREL in the Aquificales sequences for T genes than for E genes, this would imply that composition biases could be at the origin of the signal favouring the Thermotogales placement. We find that T genes in *Aquifex aeolicus *and *Sulfurihydrogenibium azorense *contain 45,4% of IVYWREL amino-acids, against 44.4% for E genes. As the difference is not significant (χ^2 ^test, p-value = 0.61), there is no evidence that the T signal is coming from compositional artefacts.

Consequently it appears that neither the signal favouring a close relationship between Aquificales and epsilon-Proteobacteria nor the signal favouring a close relationship between Aquificales and Thermotogales seem induced by a reconstruction artefact, namely long branch attraction or compositional convergence. Similarly, this suggests that the trees placing Aquificales close to Thermotogales in the whole genome study may not come from long branch attraction or compositional artefacts. Therefore, incongruences found between the T and E groups of genes probably unveil different gene histories: at least one of these two prevailing signals comes from HGTs.

### Detection of Horizontal Gene Transfers in the concatenate

We used the 181 possible Aquificales positions whose likelihoods had been computed earlier to search for evidence of HGTs affecting Aquificales genes. Because the taxonomic sampling was as exhaustive as currently possible, and because all possible positions for Aquificales among Bacteria have been tried, it is expected that few HGTs affecting Aquificales might escape this screening.

Naturally, some genes from other Bacteria present in the dataset also underwent transfers that will not be detected using our approach. But neglecting such transfers should not affect our results, since the focus of this study is the position of Aquificales,.

**Figure 5:**
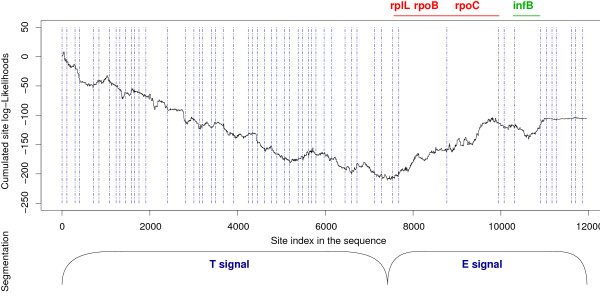
**Comparison between site likelihoods when Aquificales are placed close to Epsilon-proteobacteria and when they are placed with Thermotogales.** Upper panel: summed differences between site log-likelihoods obtained when Aquificales are placed with epsilon-Proteobacteria and when they are placed with Thermotogales. A descending trend means that a consecutive series of sites favours the Thermotogales position (T signal), whereas an ascending trend means that a series of sites favours the epsilon-proteobacterial position (E signal). Genes have been ordered according to their position along the *Aquifex *genome. Dashed blue lines represent gene boundaries. The red interval represents the genes which appear to contain most of the E signal. The green interval represents gene infB, in which the curve first decreases and then increases. Lower panel: result obtained by the Maximum Predictive Partitioning algorithm when asked to find the most likely partition of the sites in two segments. The *a posteriori *most likely model for the first segment is the tree in which Aquificales are sister group to Thermotogales, and the second segment is best fitted by the tree in which Aquificales are sister group to epsilon-Proteobacteria.

The top curve of Fig. 5 shows the cumulative sum of the log-likelihood differences between the tree in which Aquificales are close to epsilon-Proteobacteria and the tree in which Aquificales are close to Thermotogales. If asked to divide this curve, one would probably cut it in two parts, the first one decreasing, and the second one increasing. This would plead for two signals, first one in favour of the Thermotogales position, and then one in favour of the epsilon-Proteobacterial position. However, this division would be based on the comparison of only two trees, whereas 181 different positions should be compared.

We used the Maximum Predictive Partitioning (MPP) algorithm to find what are the two prevailing signals in the alignment among all 181 compared positions [43]. This algorithm identifies the best way of dividing the data in two parts and assigning each to a specific tree position. The results are displayed in the bottom panel of Fig. 5. The MPP algorithm divides the alignment very close to the site in which the curve changes from descending to ascending trends. The most likely positions affected to each of the two parts, among all 181 possible positions, are first the tree in which Aquificales are close to Thermotogales, and second the tree in which Aquificales are close to epsilon-Proteobacteria. Therefore, the two dominant signals in the alignment are T and E signals. Furthermore, the sequence concatenate was built following the gene order in the *Aquifex aeolicus *genome. Consequently, the fact that series of consecutive sites support the same phylogenetic position for *Aquifex *means that whole genes plead for each hypothesis.

**Figure 6:**
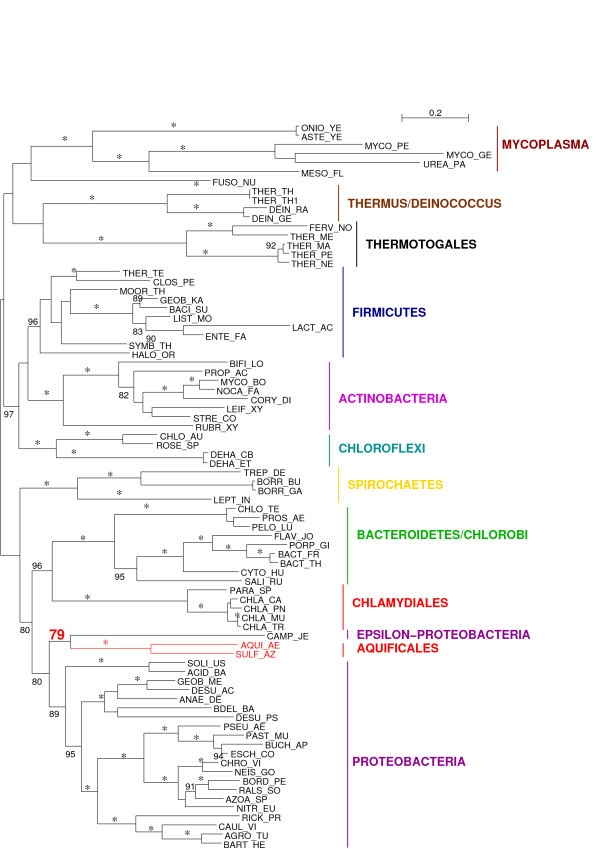
**Unrooted tree obtained from the concatenation of rplL-rpoB-rpoC.** Colors and symbols as in Fig. 3.

The issue now is to decide which of these two dominant signals is most likely HGT, and which has the highest chance of coming from vertical inheritance. One can rely on the *Aquifex aeolicus *genomic map to find the solution: if a hypothesis is favoured by an isolated island that concentrates a few genes, it is likely to be the signature of a large horizontal transfer affecting a unique region of the genome. Contrary to the T signal, the signal that favours a close relationship between Aquificales and epsilon-Proteobacteria is limited to a few clustered genes, mainly consisting of the rplL-rpoB-rpoC operon (characterized in *E. coli*, [44,45]), which seems conserved in most bacterial genomes. This clustering strongly suggests that the epsilon-proteobacterial signal comes from horizontally transferred genes, through a single transfer of the whole rplL-rpoB-rpoC operon, from epsilon-Proteobacteria to Aquificales. Indeed, if only these three genes are concatenated and submitted to phylogenetic analysis, Aquificales are found clustered with epsilon-Proteobacteria with a fairly high bootstrap support (79%, Fig. 6). As these transferred genes are large, they contribute a substantial amount of signal in the complete concatenate. This large transfer appears unexpected, since it concerns informational genes, involved in translation (rplL) and transcription (rpoB-rpoC), but it has already been suggested by Iyer, Koonin and Aravind [46]; the alternative hypothesis of the E signal being the real phylogenetic signal would require repeated HGTs of 11 genes between Thermotogales and Aquificales along all the *Aquifex *genome (Table 1), or a very large HGT of 11 genes, subsequently scattered along the Aquifex genome. Both explanations seem more unlikely. Consequently, we favour the hypothesis of a single HGT of the whole rplL-rpoB-rpoC operon from an ancestor of epsilon-Proteobacteria to Aquificales.

**Table 1:** Position of Aquificales in phylogenies built from single genes present in the concatenated alignment

*Position in the genome (locus index)*	*Gene name*	*Phylogeny: group neighbouring Aquificales*
8	rpsJ	Thermotogales
11	rplD	*Deinococcus/Thermus*
13	rplB	*Fusobacterium nucleatum*
16	rplV	*Thermoanaerobacter *tengcongensis
17	rpsC	Thermotogales
18	rplP	Planctomycetes
20	rpsQ	*Chloroflexi*
73	rpsK	Planctomycetes
74	rpsM	a clade comprising spirochaetes and *Bacteroidetes/Chlorobi*
123	rpsP	*Bdellovibrio*
226	rpsO	Planctomycetes
287	smb	Thermotogales
461	gatB	Thermotogales
609	hypothetical protein	Clostridiales
712	frr	*Chloroflexi*
735	rpsL2	Thermotogales
792	cycB1	a clade comprising *Thermoanaerobacter tengcongensis *and *Bdellovibrio*
946	rnc	Thermotogales
1478	recR	*Leptospira interogans*
1489	trmD	Thermotogales
1493	dnaG	a clade comprising Spirochaetes and Thermotogales
1645	rpsE	*Deinococcus/Thermus*
1648	rplR	Clostridiales
1649	rplF	Thermotogales
1651	rpsH	a clade comprising Thermotogales and *Deinococcus/Thermus*
1652	rplE	*Actinobacteria*
1654	rplN	*Mycoplasma*
1767	rpsT	*Proteobacteria*
1773	rpmA	*Borrelia*
1777	infC	*Leptospira interogans*
1832	rpsG1	Thermotogales
1878	rpsI	*Desulfotalea psychrophila*
1919	era2	Thermotogales
1933	rplK	Thermotogales
1935	rplA	*Chloroflexi*
1939	rpoB	*Campylobacter jejuni*
1945	rpoC	*Campylobacter jejuni*
2007	rpsB	a clade comprising Thermotogales and *Cyanobacteria*
2032	infB	a clade comprising Proteobacteria, *Bacteroidetes-Chlorobi*, Spirochaetes, Chlamydiales
2042	rplI	a clade comprising delta-Proteobacteria, *Chloroflexi*, and Planctomycetes

Results not unambiguously interpretable are not shown.

Such a hypothesis is relevant to the relative dating of Aquificales and epsilon-Proteobacteria: a transfer from an ancestor of epsilon-Proteobacteria to an ancestor of *Aquifex aeolicus *and *Sulfurihydrogenibium azorense *implies that these ancestors are contemporary. Although in trees of life obtained from rRNAs or concatenated proteins and rooted between Bacteria and Archaea-Eukaryota Aquificales are found very close to the root of Bacteria, the divergence between *Aquifex *and *Sulfurihydrogenibium *should not be more ancient than the divergence of epsilon-Proteobacteria from other Proteobacteria.

A gene-by-gene analysis adds support to the hypothesis that the dominating signal places Aquificales whith Thermotogales. Table 1 shows that, among the 39 gene phylogenies that can be unambiguously interpreted, 11 place Aquificales with Thermotogales while only 2 (RpoB and RpoC) place Aquificales with epsilon-Proteobacteria. The phylogeny of rplL is difficult to interpret, with Aquificales placed close to Delta-proteobacteria and epsilon-Proteobacteria, which might be due to the short length of this gene (139 sites). Strikingly, 13 genes place Aquificales with Gracilicutes, either close to Planctomycetes, to Spirochaetes, to Bacteroidetes-Chlorobi or to Proteobacteria. A single dominant pattern does not emerge from these gene trees: therefore they do not argue in favour of a specific relationship between Aquificales and a particular group of Gracilicutes. These results rather suggest either uncertainties in phylogenetic reconstruction or repeated horizontal gene transfers between Aquificales and various Gracilicute donors.

In conclusion, the epsilon-proteobacterial signal in the concatenated carefully chosen proteins probably derives from horizontally transferred informational genes, and the Thermotogal signal might be the signal of vertical descent. This conclusion is perfectly congruent with the results from the whole genome analysis. However, the epsilon-Proteobacterial vicinity hypothesis was originally based upon rare genomic changes. How can this hypothesis be reconciled with our conclusions?

### The impact of horizontal gene transfers on rare genomic changes

The prevailing cladistic study arguing that Aquificales should be placed as a neighbour to Proteobacteria was performed by Griffiths and Gupta [6], where inserts in 4 genes were found to support this hypothesis. These 4 genes are rpoB, rpoC, alanyl-tRNA synthetase and inorganic pyrophosphatase.

Interestingly, two of these four genes, rpoB and rpoC, are included in our concatenated alignment. Because they are clustered in the *Aquifex aeolicus *genome and display the same non-mainstream phylogenetic signal, we have diagnosed them as resulting from HGT from epsilon-Proteobacteria. Therefore, the two large inserts that Griffiths and Gupta found are no proof of a particular relatedness but rather of a HGT.

The alanyl-tRNA synthetase has not been included in our concatenate because tRNA synthetase genes are known to be extremely prone to HGT [47]. The analysis of the alanyl-tRNA synthetase gene family of the HOGENOM database (family HBG008973), confirms that this gene might not be a good phylogenetic marker. In the tree built from this family with PhyML, *Aquifex aeolicus *is found close to the spirochaete *Leptospira*, together close to Clostridiales, the Planctomycete *Rhodopirellula baltica *is found as a neighbour to Deinococcales (data not shown), among other oddities. All these relations are inconsistent with the tree built from the concatenate and inconsistent with current ideas about bacterial taxonomy. Therefore, using the alanyl-tRNA synthetase gene family to resolve bacterial phylogeny appears inadequate.

Finally, the inorganic pyrophosphatase tree as retrieved from HOGENOM (family HBG000457) shows *Aquifex aeolicus *inside Proteobacteria, close to Alpha-proteobacteria, which are not monophyletic. It appears that this gene family has undergone a duplication (Cyanobacteria are represented twice in the tree in widely separated positions) as well as horizontal gene transfers (Archaea are clustered in two groups widely separated in the tree, as well as Chlamydiales). Overall, the history of inorganic pyrophosphatase is probably too complex to be used as a marker of species relationships.

Consequently, the rare genomic changes that were used to argue for a specific relatedness between Aquificales and Proteobacteria most likely come from HGT between these two clades, as already observed in the above analyses (Fig. 1 for instance).

The fact that the outer membrane of *Aquifex *closely resembles the outer membrane of other Proteobacteria was also used [4] to argue that Aquificales are more closely related to Proteobacteria than to Thermotogales. It is unclear why this character would be particularly immune to HGT; the outer membrane most likely possesses a strong adaptive value, so that the transfer of the operational genes coding for such a structure could be positively selected and rise to fixation in a species. Given the very high rate of HGT seen in *Aquifex *genome, it is not unreasonable to assume that the proteobacterial type of outer membrane might have been transferred to Aquificales. Similarly, the close relationship found between epsilon-Proteobacteria and Aquificales in trees based on cytochromes b and c might also come from a HGT of a whole operon, as concluded by Schutz *et al*. [48]. On the contrary, our counting analysis confirms that informational genes are less prone to HGT than operational genes, and their signal clusters Aquificales and Thermotogales.

### Further difficulties to resolve the tree of Bacteria

A possible approach to uncover a putative species tree of Bacteria, or at least a tree for a core set of bacterial genes, would be to remove transferred genes from a dataset, concatenate all genes that have not been detected as having been transferred, and use them to build a phylogenetic tree. Such an approach would be expected to yield better trees, with higher bootstrap supports. However, the phylogeny obtained on the concatenate in the same conditions as before (without recoding) but after removal of the rplL-rpoB-rpoC genes does not show a significantly better support for most of its nodes than the phylogeny shown in Fig. 3 (average bootstrap support for the tree without the three genes, 90.9, and for the tree with all genes, 90.3; p-value = 0.17 with a Student paired t-test, p-value = 0.288 with a Wilcoxon signed rank test). This is probably due to the fact that bootstrap supports increase with the number of characters; the length parameter therefore counters the expected positive effect associated with the removal of discordant signal. Topologically, both trees are highly congruent, with the main noticeable difference being the placement of *Fusobacterium nucleatum*, which leaves its position as sister-group to Thermotogales and Aquificales in Fig. 3 to nest inside the Firmicutes as a sister group to *Mycoplasma*. This placement might stem from a long branch attraction, as both *Mycoplasma *and *Fusobacterium *have long terminal branches, or alternatively might reveal the true history of *Fusobacterium nucleatum*, as suggested by Mira and co-workers [49]. Certainly this organism deserves further study, possibly with techniques such as those that were used in this article.

It is interesting to note that the removal of genes thought to have been transferred has not improved the phylogeny. A most promising avenue for further research in deep phylogenies would probably involve the development of models explicitly taking into account HGT, as proposed by Suchard [50] or, in other contexts, by Edwards, Liu and Pearl [51,52] and Ané *et al*. [53]. HGTs should be modelled as a genuine biological phenomenon on equal footing with vertical descent to represent the evolution of bacterial genomes. The resulting species tree would correspond to the history of those genome parts that have been vertically inherited at any time during evolution. The vertically inherited portions of a genome at a given time need not be vertically inherited at all time, so that a species tree could be inferred as long as, at any time, some vertical signal could be recovered.

Another additional difficulty might be that the gene is not necessarily the atomic unit of transfer: transfers may affect only parts of a gene, through recombination. In this respect, the analysis of Figure 5 reveals a striking pattern in the Initiation Factor 2 gene (infB, green line). In this large gene (the *Aquifex aeolicus *protein is 805 amino-acids long), the curve of the difference in log-likelihoods between the epsilon-proteobacterial and the thermotogal positions of Aquificales first decreases for about half its length, and then increases. This pattern is suggestive of a recombination event inside the gene.

**Figure 7:**
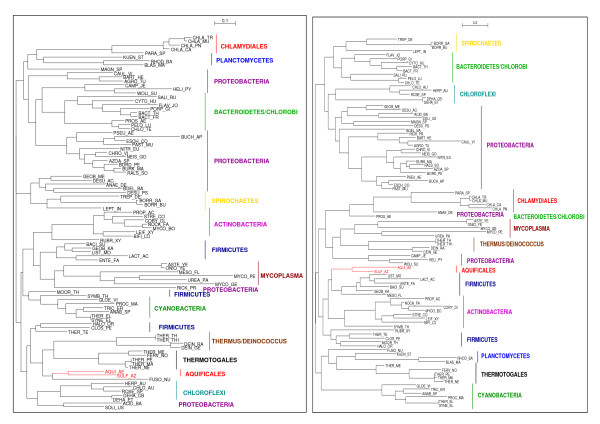
**Unrooted trees corresponding to the infB gene.** Left: tree corresponding to the first 301 sites. Right: tree corresponding to the remaining 246 sites. Colors as in Fig. 3.

To test for recombination, we divided the infB alignment in two at the point where this curve changes trend and built phylogenetic trees for both partial alignments (Fig. 7). In the first resulting tree, Aquificales plus *Fusobacterium nucleatum *make together a sister group to Thermotogales plus *Deinococcus/Thermus*. In the second tree, Aquificales are a sister group to a subclade of Firmicutes. These two branchings are consistent with the slope of the curve of Fig. 5, first descending, as Aquificales are close to Thermotogales, and then ascending, as Aquificales are far from Thermotogales. To assess whether the differences in the topologies were significant, Consel was used [42] on these last two trees. The first part of the alignment strongly rejected the tree obtained for the second part (AU test p-value: 4.10^-36^; SH and KH p-value: 0), and *vice versa *(AU test p-value: 1.10^-06^; SH and KH p-value: 0). Therefore a strong signal for recombination within the gene infB is found, possibly between Firmicutes and Aquificales.

This indicates that the unit of transfer between Bacteria is not necessarily the gene, but can also be parts of a gene. Models aiming at resolving the bacterial tree may need to take this additional complexity into account.

## Conclusion

Overall, the signal in favour of a close relationship between Aquificales and epsilon-Proteobacteria has been shown to be coming from a lateral transfer and not vertically inherited, both in protein phylogenies and in cladistic analyses. A large HGT involving three consecutive genes encoding two RNA polymerase subunits and a ribosomal protein has been detected. This large gene transfer between epsilon-Proteobacteria and Aquificales can be understood in terms of a shared ecological niche: some epsilon-Proteobacteria are indeed found in hyperthermophilic environments [54].

The present single-gene analyses suggested that gene transfers may have frequently occurred between Aquificales and various Gracilicutes and Proteobacteria in particular, which explains why cladistic analyses of rare genomic changes or of domain contents often place *Aquifex *inside Gracilicutes.

Bacterial phylogeny is crucial to understand the evolution of the biosphere, as it provides a backbone permitting to integrate the evolution of life as revealed from molecular phylogenies with the history of the earth, as dug up by geology. There is no doubt that HGT has played a major role in the evolution of Prokaryotes, to the point that there might be no gene that has never undergone HGT; however a few gene families may have seldom been transferred, and they might bear sufficient signal to unveil the vertical history of the genome, provided powerful computational methods modelling both gene transfers and intra-genic recombination are developed.

Nonetheless, because Aquificales are often found grouped with Thermotogales, and because this phylogenetic signal does not seem to result from known artefacts such as long branch attraction or compositional bias, if there is a species tree in Bacteria, Aquificales are to be considered as a sister group to Thermotogales. This clarification does not dramatically affect the scenario for the evolution of life proposed by Cavalier-Smith [9], except that Aquificales diverged earlier than proposed. However the present results question the methodology used to build this scenario because the rare genomic changes method requires that HGT does not affect used marker genes. In the case of the Aquificales, we have shown that this requirement is not fulfilled.

## Methods

### Whole phylome analysis

In order to get a whole genome view of Aquificales phylogenetic relationships, we queried the HOGENOM database (release 03, October 2005) using the TreePattern program in FamFetch [55]. HOGENOM is a database that clusters sequences from whole genomes into homologous gene families, and builds trees based on these families with PhyML using a gamma law with 4 classes of substitution rates, with estimated alpha parameter and proportion of invariable sites. Trees corresponding to all 892 families in which there was a sequence from *Aquifex aeolicus *were automatically analysed, and each sequence from *Aquifex *was classified according to what group of species appeared as its closest neighbour, not taking into account branch support or branch length. This gave counts of *Aquifex *genes found close to Thermotogales, Firmicutes, epsilon-Proteobacteria, *etc*... Cases where *Aquifex *genes were found close to a non-monophyletic group of species were discarded, which left 578 gene trees. These counts were further classified into two functional categories, "informational genes" and "non-informational genes", through TIGRFAM annotations [56]. A functional category could be determined for 351 families. "Informational genes" were genes classified in TIGRFAMs whose function was part of "Transcription", "DNA metabolism", "Protein synthesis"; "non-informational genes" were those whose role was part of other major functional classes.

### Concatenate assembly

Nearly universal gene families which had only one copy per genome were used to minimize problems of ill-defined orthology. Consequently, gene families from the HOGENOM database of families of homologous genes (release 03, October 2005) that displayed a wide species coverage with no or very low redundancy in all species were selected. This provided 70 gene families. Sequences from representative genomes from Archaea were retrieved from these families, and sequences from genomes not present in the release 03 of HOGENOM but whose phylogenetic position was interesting were included in the families. These studied genomes are listed in Additional files 1, 2 and 3 and were downloaded from the Joint Genome Institute [57], The Institute for Genomic Research [58] or the National Center for Biotechnology Information [59], and were searched for homologous genes using BLAST [60]; only the best hit was retrieved. The gene families were subsequently aligned using MUSCLE v3.52 [61] and submitted to a phylogenetic analysis using the NJ algorithm [62] with Poisson distances as implemented in Phylo_Win [63]. During this step, families in which there seemed to be a gene transfer between a bacterial species and Archaea were discarded, as well as amino-acid synthetases, which are known to be prone to HGT [47]. In the rare families where there were two sequences from the same species, the sequence showing the largest terminal branch length or whose position was most at odds with the NCBI classification was discarded. This whole process provided 56 gene families and 94 bacterial species. Only bacterial sequences were used in the rest of the study, because our focus is on the bacterial phylogeny itself. The 56 families were submitted to Gblocks [64] to discard parts of the alignments that were unreliable, but using a non-stringent site selection, because the subsequent analyses should permit to sort biased from genuine signal. Consequently, the following Gblocks parameters were used: the minimum numbers of sequences used to define a conserved or a flanking position were set at 50% of the total number of sequences, the minimum length of a block was set at 2 sites, and all positions could be kept by the algorithm, even if they contained gaps. The resulting alignments were then concatenated using ScaFos [65], following the order of genes along the *Aquifex aeolicus *genome. The amount of missing data was low, reaching 21% at its maximum in *Thermotoga petrophila*.

### Phylogenetic analyses

A phylogenetic tree was built from the concatenate under the Maximum Likelihood criterion using PhyML v.2.4.4 [66] with the JTT model [67], and a discretized gamma law with 4 categories to model evolutionary rate variation. This first tree was used to compute site-specific evolutionary rates using BppML from the Bio++ package [68], which allowed for the removal of saturated sites. A new tree was built using this refined alignment, with the same parameters plus an estimated proportion of invariant sites and with a non-parametric bootstrap analysis (200 replicates), and was used as a reference for the rest of the work. An estimated proportion of invariant sites was not used in the previous analysis because it had not been implemented in the used version of Bio++. Noticeably, the topology was found to be unchanged when Aquificales were removed from the alignment and the tree re-computed. Similarly, the topology was nearly identical when two free-living espsilon-Proteobacteria (*Sulfurovum *NBC37-1 and thermophilic *Nitratiruptor *SB155-2 [40],) were added, and the tree recomputed with PhyML v3.0; for this tree, the minimum of SH-like and chi2-based support was computed instead of bootstrap support [69]. An additional test was performed to assess the impact of compositional heterogeneity as well as saturation: the alignment without saturated sites was recoded in 4 categories [70,37]. In this recoding, aromatic (FWY) and hydrophobic (MILV) amino-acids were grouped in a single state, basic amino-acids (HKR) in another, acidic (DENQ) amino acids in one more state, and the fourth state contained all other amino acids (AGPST) to the exception of cysteine which was coded as missing data. The recoded alignment was subjected to a phylogenetic analysis with the GTR model [71], an estimated proportion of invariant sites, a gamma law discretized in 8 categories with its alpha parameter estimated, and 200 bootstrap replicates.

The tree without the Aquificales was used as a scaffold upon which all possible Aquificales positions were tried in turn. The likelihoods for each of these positions were computed using BppML from the Bio++ package. Evolutionary rates per site as well as likelihoods per site were simultaneously inferred. Site evolutionary rates were obtained by computing the average of the gamma law rate categories weighted by their posterior probabilities.

The tree containing only the rplL-rpoB-rpoC genes was obtained with PhyML as described above and with a non-parametric bootstrap analysis based upon 500 replicates.

Individual gene trees were built using PhyML with the same parameters as above except that the gamma law was discretized in 8 categories.

### Concatenate segmentation and HGT identification

We wanted to know which was the most likely segmentation in two segments of the alignment according to site likelihoods for all topologies. It was computed using Sarment [72] with the Maximum Predictive Partitioning algorithm [43]. This algorithm was input a matrix containing the site log-likelihoods for all 181 topologies tested (obtained by placing the Aquificales in all possible positions in the backbone bacterial phylogeny) and for the whole alignment. The best log-likelihood of a given segmentation is the sum of the best log-likelihoods of its segments, that are computed as follows: on a segment, for each of the 181 topologies tested, the log-likelihood of a topology is the sum of all site log-likelihoods on the alignment. This procedure produces 181 log-likelihoods, the maximum of which is the best log-likelihood of this segment. Once this maximum is found, it clearly associates a most likely topology to each segment of the alignment. All statistical analyses were done with the seqinR package [73] in R [74].

## Abbreviations

HGT: Horizontal Gene Transfer; rRNA: ribosomal Ribo-Nucleic Acid; indel: insertion-deletion; MPP: Maximum Predictive Partitioning.

## Authors' contributions

MG and BB designed the study. LG performed the segmentation analysis, and BB performed the other experiments. BB wrote most of the manuscript, which was improved by LG and MG.

## Supplementary Material

Additional file 1 The list of species used in the study, and their abbreviated names as found in the figures of the article. Click here for file

Additional file 2 Unrooted phylogenetic tree of Bacteria obtained after the addition of two free-living epsilon-Proteobacteria, *Sulfurovum *NBC37-1 and thermophilic *Nitratiruptor *SB155-2. Click here for file

Additional file 3 The list of 56 HOGENOM gene families used to estimate species trees, with the corresponding function description. Click here for file
