# Supplementary figures and images for: Accounting for horizontal gene transfers explains conflicting hypotheses regarding the position of aquificales in the phylogeny of Bacteria
Source: BMC Evol Biol. 2008 Oct 3;8:272. doi: 10.1186/1471-2148-8-272 (PMC2584045; doi:10.1186/1471-2148-8-272)

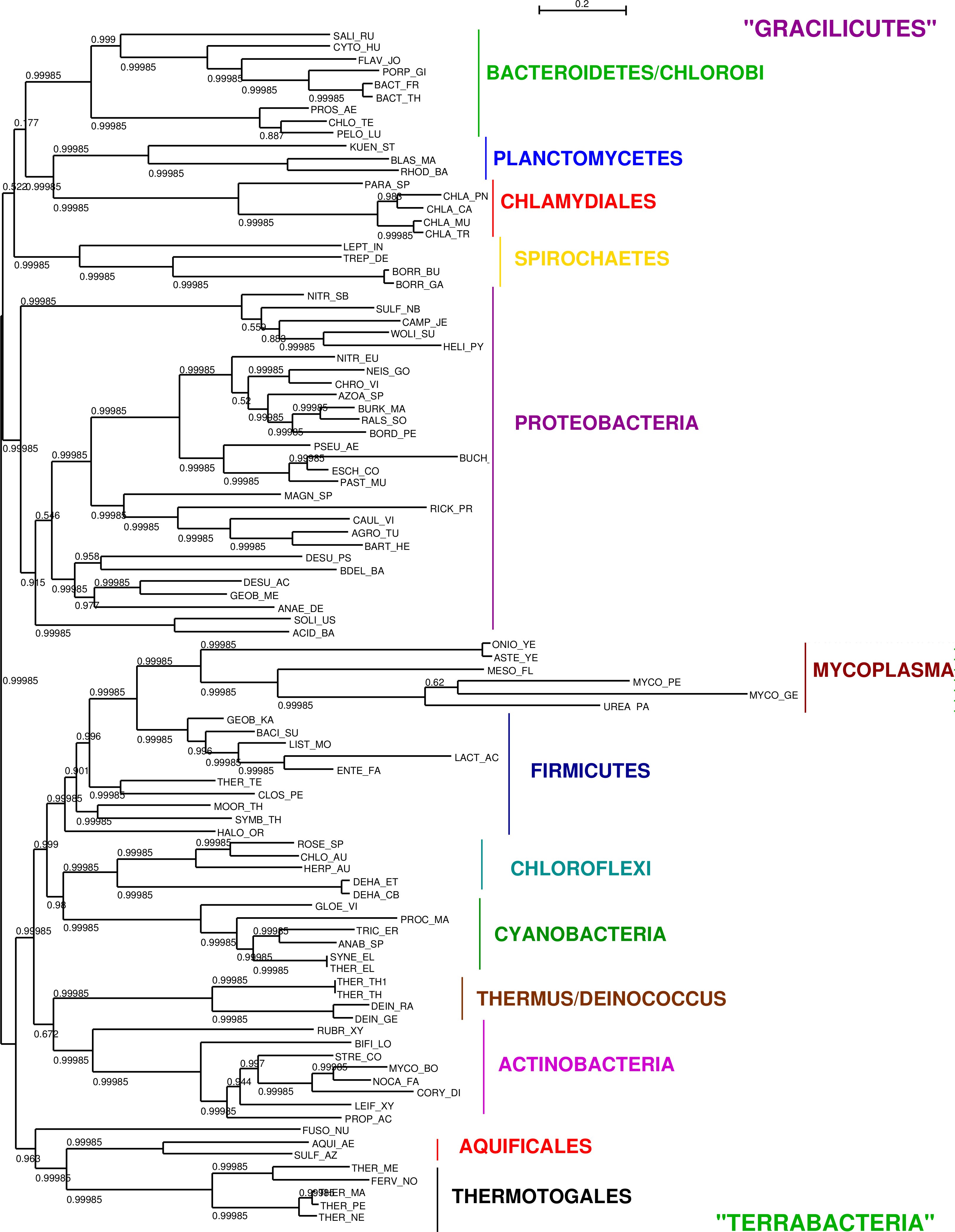

Supplement: Additional file 2 — Unrooted phylogenetic tree of Bacteria obtained after the addition of two free-living epsilon-Proteobacteria, Sulfurovum NBC37-1 and thermophilic Nitratiruptor SB155-2. [file 1471-2148-8-272-S2.jpeg]
